# Precision synbiotic intervention with 2′-fucosyllactose and infant-derived *Bifidobacterium* modulates gut-immune axis and reduces disease risk in toddlers: a randomized controlled trial

**DOI:** 10.3389/fnut.2026.1856180

**Published:** 2026-07-30

**Authors:** Ke Chen, Xi Zhang, Shanshan Jin, Jiayi Zhong, Haixia Chen, Genquan Yue, Yonghui Cheng, Rufeng Peng, Ziji Fang, Feitong Liu, Zerong Lu, Lingling Zhao, Ruibiao Hu, Xiaohan Tao, Qilin He, Changqi Liu

**Affiliations:** 1 Department of Clinical Nutrition, Chengdu Women’s & Children’s Central Hospital, School of Medicine, University of Electronic Science and Technology of China, Chengdu, China; 2 Baoxing Center for Disease Control and Prevention, Ya’an, China; 3 Longmen Town Central Health Center, Ya'an, China; 4 Baoxing Maternal and Child Health Care Hospital, Ya’an, China; 5 School of Medicine, University of Electronic Science and Technology of China, Chengdu, China; 6 H&H Group, China Research and Innovation Center, Guangzhou, China; 7 School of Food Science and Engineering, South China University of Technology, Guangzhou, China; 8 Graduate School of International Business, the Catholic University of Korea, Bucheon, Republic of Korea; 9 School of Exercise and Nutritional Sciences, San Diego State University, San Diego, CA, United States

**Keywords:** 2′-fucosyllactose, *Bifidobacterium*, gut microbiota, mucosal immunity, synbiotics

## Abstract

**Background:**

Early childhood represents a critical window for nutritional programming of immune function and microbiome establishment. While human milk oligosaccharides (HMOs) and probiotics individually demonstrate health benefits, their synergistic integration as synbiotics remains underexplored in toddler populations. This study aimed to evaluate whether a precision synbiotic combining 2′-fucosyllactose (2’-FL) with infant-adapted *Bifidobacterium* strains provides superior protection against common pediatric conditions compared with probiotics alone or placebo, and to elucidate underlying gut-immune mechanisms.

**Aim:**

To determine the efficacy of a 2’-FL-containing synbiotic on infectious and atopic disease incidence, gut microbiota composition, and intestinal immune markers in children aged 1–3 years.

**Methods:**

In this multicenter, doubleblind, placebo-controlled, three-arm trial, 390 healthy toddlers were randomized (1:1:1) to receive (1) synbiotic (2’-FL 1.0 g/day + *B. infantis* R0033 1.5 × 10^9^ CFU + *B. bifidum* R0071 1.5 × 10^9^ CFU); (2) probiotic (identical strains/doses); or (3) placebo (maltodextrin 1.5 g/day) for 12 weeks, with 12-week follow-up. Primary outcome was upper respiratory tract infections (URTIs) incidence over 24 weeks. Secondary outcomes included pneumonia, diarrhea, eczema, antibiotic use, gut microbiota (16S rRNA V3-V4 sequencing), and fecal immune biomarkers (calprotectin, sIgA, HBD-2, LL-37). Dietary intake was monitored to control nutritional confounders.

**Result:**

Synbiotic vs. placebo: URTI incidence reduced from 85.4 to 55.4% (adjusted risk ratio (aRR) 0.64, 95% CI 0.43–0.95; ARR 30.0%, NNT = 3.3; *p* = 0.028); pneumonia from 17.7 to 7.7% (RR 0.40, 0.17–0.94; *p* = 0.034); diarrhea from 17.7 to 10.0% (aRR 0.54, 0.30–0.95; *p* = 0.032); and eczema from 13.1 to 1.5% (aRR 0.12, 0.04–0.35; ARR 11.6%, NNT = 2.9; *p* < 0.001). Probiotic alone reduced only eczema (aRR 0.10, 0.03–0.40; *p* = 0.001). The synbiotic significantly altered gut beta-diversity (Bray-Curtis, *p* = 0.042) and enriched *Bifidobacterium catenulatum* and *B. kashiwanohense* species with conserved HMO utilization pathways. At 24 weeks, synbiotic reduced fecal calprotectin (62.7 ± 23.9 vs. 77.9 ± 24.6 μg/g; *p* = 0.004) and increased sIgA (1.37 ± 0.38 vs. 1.15 ± 0.49 mg/g; *p* = 0.014), indicating enhanced intestinal immune homeostasis.

**Conclusion:**

A 12-week precision synbiotic intervention combining 2’-FL with infant-derived *Bifidobacterium* strains significantly reduced the burden of respiratory infections, diarrhea, and atopic dermatitis in toddlers, with effects exceeding probiotics alone. These benefits were mediated by targeted gut microbiota modulation and enhanced mucosal immune function. This study provides evidence for integrative nutritional strategies in early childhood disease prevention and supports the development of next-generation synbiotic formulations.

**Clinical trial registration:**

This study was registered in the Chinese Clinical Trial Registry (ChiCTR2400088943) prior to enrollment (https://www.chictr.org.cn/showprojEN.html?proj=235745).

## 1 Introduction

Early childhood represents a critical window for growth, immune system development and the establishment of the gut microbiota. During this period, infants and toddlers are highly susceptible to common diseases, including respiratory tract infections (RTIs), gastrointestinal infections, allergic disorders, and functional gastrointestinal disturbances (1–3). These conditions not only impose a significant burden on child health and healthcare systems but may also have long-term negative consequences for physical growth, neural development, and immune programming (4). Preventive strategies that are safe, effective, and biologically plausible are therefore of substantial clinical and public health importance.

The gut microbiota plays a central role in shaping immune development during early life. The first three years of life are characterized by rapid microbial succession, during which the gut ecosystem transitions from a relatively simple community to a more complex and stable adult-like microbiota. This early microbial colonization influences immune tolerance, intestinal barrier integrity, and host metabolism (5, 6). Disruptions of gut microbial development, commonly referred to as dysbiosis, have been associated with an increased risk of infectious diseases, allergies, metabolic disorders, and chronic inflammatory conditions in later life (7). Consequently, preventive measures that modulate the gut microbiota during infancy and early childhood are considered promising approaches to enhance immune resilience and reduce disease susceptibility.

Human milk is widely recognized as the optimal source of nutrition for infants and contains a diverse array of bioactive components, among which human milk oligosaccharides (HMOs) are the third most abundant solid component after lactose and lipids. HMOs are indigestible by the infant but selectively utilized by beneficial gut microbes, particularly *Bifidobacterium* spp., thereby shaping the early gut microbiota (8). Among HMOs, 2′-fucosyllactose (2’-FL) is one of the most abundant and extensively studied structures. Evidence suggests that 2’-FL promotes the growth of *bifidobacterium*, inhibits pathogen adhesion to the intestinal epithelium, modulates epithelial barrier function, and exerts immunomodulatory effects through host–microbe interactions (9). Clinical studies have demonstrated the safety and tolerability of 2’-FL supplementation in infant formulas, with potential benefits for immune-related outcomes; however, evidence in toddlers and older infants remains limited (10).

Probiotics, defined as live microorganisms that confer a health benefit on the host when administered in adequate amounts, have been extensively studied in pediatric populations (11). *Bifidobacterium* genus are dominant members of the infant gut microbiota and play a particularly important role in immune development, pathogen exclusion, and metabolic modulation. Specific *Bifidobacterium* species have been shown to reduce the incidence and duration of gastrointestinal and respiratory infections, alleviate allergic manifestations, and improve gut barrier integrity in infants and young children (12). Importantly, probiotic effects are strain-specific and may be influenced by the availability of fermentable substrates in the intestinal environment (13).

The concept of synbiotics, defined as combinations of prebiotics and probiotics that act synergistically, has gained increasing attention in recent years (14). HMOs such as 2’-FL may serve as selective substrates for bifidobacteria, enhancing their colonization, persistence, and metabolic activity in the gut (15). Animal studies suggested that combined administration of HMOs and *Bifidobacterium* strains may lead to greater modulation of gut microbial composition, increase production of short-chain fatty acids (SCFAs), improve mucosal immunity, and enhance protection against pathogens compared with either component alone (16). However, clinical evidence supporting these synergistic effects in young children, especially those aged 1 to 3 years old, remains scarce.

Given the high prevalence of common childhood conditions (17–19), the pivotal role of gut microbiota in immune maturation, and the promising but incomplete evidence regarding HMO-probiotic synergy, there is a strong scientific rationale to investigate the combined effects of 2’-FL and *Bifidobacterium* strains in early childhood. In particular, the potential benefits of a dual *Bifidobacterium* strain formulation combined with 2’-FL on common disease prevention and gut microbiota development have not been adequately explored in well-designed clinical studies (20).

Therefore, we conducted a randomized, placebo-controlled, blinded trial in several communities in Sichuan Province to evaluate the preventive effects of combined supplementation with 2’-FL and a dual *Bifidobacterium* strain formulation on the incidence of common diseases in young children, as well as to assess its impact on gut microbiota composition and function. We hypothesized that young children supplemented with 2’-FL combined with dual *Bifidobacterium* strains would show reduced morbidity of respiratory, gastrointestinal, and allergic diseases compared with those receiving placebo, accompanied by beneficial changes in gut microbiota and improvements in intestinal immunity.

## 2 Methods

### 2.1 Study design and participants

This randomized, double-blind, placebo-controlled, three-arm parallel trial was conducted at Baoxing Center for Disease Control and Prevention, Baoxing county and Longmen Town Central Health Center, Lushan County, Ya’an City (September 2024–September 2026). The protocol followed CONSORT 2010 and SPIRIT guidelines, was approved by the Baoxing Center for Disease Control and Prevention Ethics Committee (No. 2024–02), and registered in the Chinese Clinical Trial Registry (ChiCTR2400088943) prior to enrollment. Written informed consent was obtained from parents/legal guardians. This study is reported in accordance with CONSORT standards for randomized trials of nutritional interventions.

The inclusion criteria were:

1. Young children with aged 1–3 years old;
2. Full term birth at 37 to 42 weeks with birth weight greater than 2,500 g and less than 4,000 g, without sex restriction;
3. Formula feeding from birth to enrollment or weaning at least for 3 months before recruiting;
4. Parents voluntarily choosing to feed their children with prepackaged milk/fresh milk without probiotics and/ or prebiotics;
5. Parents or guardian agreeing to collect stool samples during the study;
6. No any allergic diseases diagnosed by clinicians (including but not limited to eczema, asthma, allergic proctocolitis, allergic rhinitis, hay fever, food allergy, etc.);
7. Having been registered in local maternal and child health hospital;
8. Parents or guardian committing not to add additional probiotics and/ or prebiotics products during the study;
9. Parents or guardian agreeing to participate in the study and signing a written informed consent.

The exclusion criteria were:

1. History of asphyxia or neonatal intensive care unit (NICU) hospitalization at birth;
2. Congenital defects or anomalies;
3. Obstetric risk factors during pregnancy such as gestational hypertension syndrome, eclampsia, gestational diabetes, cholestasis of pregnancy;
4. Use of antibiotics within 2 weeks prior to enrollment;
5. Diseases affecting growth and development within the month prior to enrollment, including pneumonia, severe diarrhea, severe constipation, malnutrition, gastrointestinal surgery, epilepsy, cerebral palsy, intellectual disability, genetic metabolic diseases, chromosomal disorders, and genetic diseases;
6. Use of experimental drugs or participation in other clinical studies prior to screening;
7. Use of the same intervention products (IPs) within one month before enrollment;
8. Use of glucocorticoids or immunosuppressants;
9. Known allergies to any components of the IPs used in this study;
10. Any other reasons deemed inappropriate for participation by the investigators, such as factors that could affect efficacy evaluation or poor compliance.

The withdrawal criteria were:

1. Erroneous inclusion or misdiagnosis;
2. Lack of clinical records for evaluation;
3. Allergic reactions to ingredients in the IPs during the trial;
4. Inability to ingest the IPs through the digestive tract;
5. Worsening of the participant’s condition during treatment requiring admission to the pediatric intensive care unit (PICU).

The study protocol was reviewed and approved by the Ethics Committee of the Baoxing Center for Disease Control and Prevention [Ethics Approval No.: Scientific Research Ethics Review No. 2024 (02)]. All parents/guardians were informed of the study details, pecific objectives related to benefits and harms, and signed the informed consent form. This study was registered in the China Clinical Trial Center (No: ChiCTR2400088943)^1^.

### 2.2 Randomization and blinding

A biostatistician who was not directly involved in conducting the study used the RAND function in Excel to generate random numbers and was the only one accessed to the random allocation sequence. A total of 390 children meeting the inclusion criteria were randomly assigned to one of the three groups: the combination of 2’-FL and probiotic intervention group (HPG), the probiotic intervention group (PG), and the placebo control group (CG) (130 children for each group). The three IPs were similar in appearance, taste, and smell, and were packaged in identical sachets with identical labeling, differing only in the subject-specific randomization number. The parents/guardians, clinicians, laboratory personnel, data manager, and statistician remained blinded to group assignments until completion of the data analysis.

### 2.3 Sample size

The sample size of the present study was determined with reference to one of our previously published trials (21) evaluating the preventive effects of nutritional interventions on RTIs. In a comparable multicenter, randomized controlled trial assessing the impact of lactoferrin supplement on children’s RTIs morbidity, approximately average 100 children were enrolled per group, and this sample size was shown to be sufficient to detect statistically significant differences in the incidence of respiratory-related illnesses between intervention and control groups. Allowing for an estimated 20% loss to follow-up, the sample size for the CG, PG and HPG was set at 130 participants each with the total number of 390.

### 2.4 Intervention

All eligible children were randomly assigned to the HPG, IG or CG. The children in HPG received a daily supplement of 1.5 billion CFU each of *B. infantis* R0033 and *B. bifidum* R0071 and 1.0 g of 2’-FL (Production No: 87-BCP-20027); the children in PG only received a daily supplement of the same strains with same CFU (Production No: 87-BCP-20028); and the children in CG received a daily supplement of 1.5 g of maltodextrin (Production No: 87-BCP-20029). All the IPs were provided by Biostime (Guangzhou) Health Products Co., Ltd. The IPs could be taken directly or mixed with warm water below 45 °C, milk, rice paste, or other liquid foods. All subjects were supplemented for 3 consecutive months and then followed up for another 3 months.

The three IPs were similar in appearance, taste, and smell, and were packaged in identical sachets with identical labeling, differing only by the subject-specific randomization number. If a child vomited within 30 min of consuming a sachet, an additional dose was administered (with a maximum of one extra dose allowed within 4 h). Dosing and re-dosing were recorded in a case report form (CRF) by the treating physicians. During the intervention and follow-up periods, any illnesses were treated by a pediatrician. If IPs and oral antibiotics were used simultaneously, a minimum interval of 3 h was maintained between the two.

### 2.5 Data collection

After enrollment, study staff performed assessments, recorded data in the CRF, and collected stool samples in accordance with the protocol. Parents and primary caregivers, responsible for the children, were administered a questionnaire by a trained on-site health worker approximately for 30 min. The questionnaire included demographic and family socioeconomic information.

The primary outcome was the frequency of upper respiratory tract infections (URTIs) during the 6-month study period. Diagnosis, treatment, and clinical management of URTIs were determined according to the relevant guidelines of the Chinese Medical Association (22).

The secondary outcomes included episodes of lower respiratory diseases, defined as the occurrence of symptoms or signs related to the lower respiratory disease within a specific period (23); episodes of gastrointestinal diseases, defined as a group of diseases affecting the function of the gastrointestinal tract, typically presenting with symptoms such as vomiting, diarrhea, constipation, or abdominal distension (24, 25); episodes of the allergic eczema, according to the diagnostic criteria for eczema (26) and gut microbiome profiles and as well as stool-based biochemical indicators, including calprotectin, secretory immunoglobulin A (sIgA), human β-defensin 2 (HBD-2) and human cathelicidin LL-37 (LL-37). Clinicians used the CRF to record the duration, frequency, therapeutic drugs used, and related symptoms and signs of respiratory, gastrointestinal, and allergic eczema within 6 months from the start of the probiotic intervention.

### 2.6 Sample collection

Fecal samples were collected at baseline, 3 month and 6 month in fecal collection tubes containing RNAlater™ solution and glass beads by the parents/guardians for microbiota analysis. All tubes were sealed tightly and stored at −80 °C until further analysis.

### 2.7 Fecal sample microbiota analysis

Total genomic DNA was extracted from the fecal samples using the CTAB/SDS method with a QIAamp Fast DNA Fecal Mini Kit (Qiagen, Valencia, California, USA) according to the manufacturer’s instructions. The bacterial 16S rRNA gene V3-V4 region was amplified using the TransGen AP221-02 Kit (TransGen, Beijing, China) with the 16S V3-V4 primers 341F-806R. Sequence analysis of the 16S rRNA amplicons was performed using Uparse software (v7.0.1001) and Quantitative Insights Into Microbial Ecology (QIIME, v1.9.1). Sequences with ≥97% similarity were clustered into operational taxonomic units (OTUs), and a representative sequence from each OTU was selected for taxonomic annotation by Silva database (SSU 138.1) using the RDP classifier. Alpha diversity was assessed using Shannon, Simpson, Chao1, and ACE indices, while beta diversity was analyzed using principal coordinate analysis (PCoA) based on Bray-Curtis distances. The differential abundance of taxa between groups was analyzed using the Kruskal-Wallis test.

### 2.8 Fecal sample biochemical analysis

Biochemical indicators in stool were determined using human protein-specific enzyme-linked immunosorbent assay (ELISA) kits (Shanghai Enzyme Linked Biotechnology Co., Ltd., Shanghai, China), following the manufacturer’s instructions. Protein concentrations were calculated based on standard curves generated using protein standards and reported in arbitrary units per milliliter.

### 2.9 Compliance

Compliance to the IPs was monitored using recording tables by parents or caregivers. Distribution (performed by field workers) and consumption of the IPs took place under close supervision. Information on the acceptability of the IPs was obtained by a short questionnaire after administration. Every weekend, field workers visited each infant’s family to examine and review the quantity of IPs consumed.

### 2.10 Statistical analysis

The primary outcome analysis was performed using both the Intention-To-Treat (ITT) and Per-Protocol (PP) dataset. Data distributions were assessed for normality using the *Kolmogorov–Smirnov* goodness-of-fit test. Normally distributed data are expressed as mean ± standard deviation, and skewed data as median (25th and 75th percentiles). All statistical tests were two-tailed, and *p* < 0.05 was considered statistically significant. The *χ^2^* test was used for inter-group comparisons of categorical clinical and demographic variables. To compare intervention effects among three groups, multiple comparisons of parameter changes after the intervention were performed using the *Student–Newman–Keuls* test for normally distributed and homogeneous data (fecal biochemical index), or the *Kruskal-Wallis* rank test for skewed data (days with common symptoms during the trial). The *Wilcoxon* rank-sum test was employed to assess inter-group differences in species relative abundance between the two pair each group.

Negative binomial regression was employed to estimate the relative risk (RR) and corresponding 95% confidence intervals (CIs) for episodes of respiratory, gastrointestinal, and allergic diseases over the 6-month study period. Binary multivariate logistic regression was also conducted to evaluate the effect of the intervention on the incidence of these diseases during the same 6-month period, with adjustment for potential confounding covariates. Statistical analyses were conducted using IBM SPSS Statistics 29.0.2 for Mac.

## 3 Results

### 3.1 Baseline characteristics

A total of 390 eligible children were enrolled in the study and randomly assigned to one of three intervention groups (*n* = 130 per group). There were no losses in follow-up, and all participants completed the CRF and were included in ITT analysis. However, 28 infants were excluded from the PP analysis due to significant protocol deviations, among whom 9 cases in the CG, 11 cases in the PG and 8 cases in the HPG were attributed to changing IPs for non-medical reasons. The compliance rate was 93.1% (121/130) in the CG, 91.5% (119/130) in the PG and 93.8% (122/130) in the HPG. Consequently, the PP data-set included 362 participants: 121 in the CG, 119 in the PG, and 122 in the HPG.

**Figure 1:**
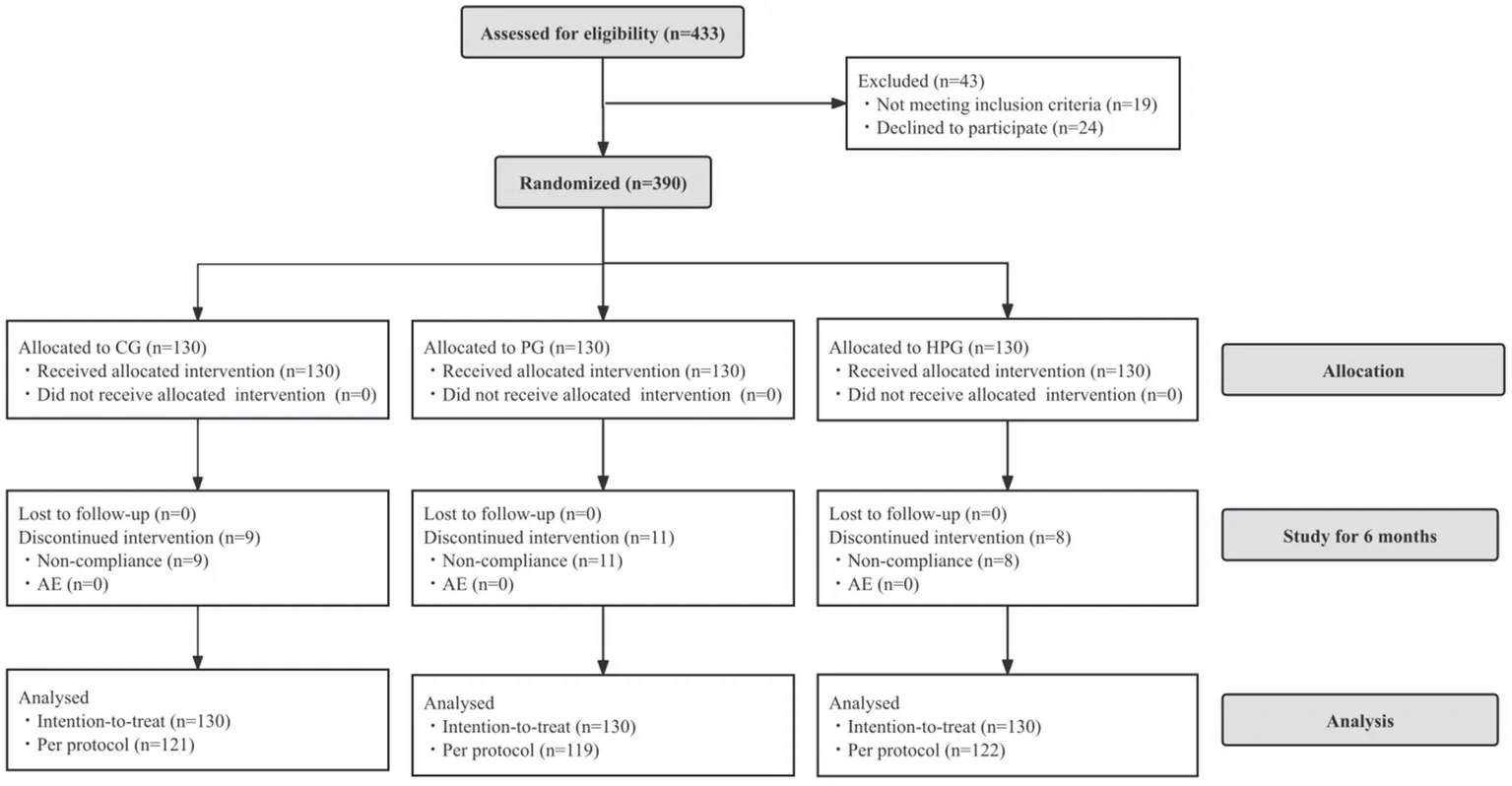
Flowchart of subject enrollment and study progress. CG, the placebo control group; PG, the probiotic intervention group; HPG, the combination of 2′-fucosyllactose and probiotic intervention group; AE, adverse events.

No adverse events related to the IPs were reported throughout the study. Figure 1 showed the flowchart for participant recruitment, allocation, follow-up, and analysis. At baseline, there were no significant differences among the three groups in terms of sex distribution, birth weight, parental education level, household per capita income, registered residence, or numbers of household residents (*p* > 0.05, Table 1).

**Table 1:** Basic clinical and demographic information of the three groups [means ± SD].

Items	CG (*n* = 130)	PG (*n* = 130)	HPG (*n* = 130)	*F/χ^2^* values	*p* values
Sex, [boy *n* (%)]	82 (63.1%)	70 (53.8%)	64 (49.2%)	5.230	0.073
Gestational age(kg)	39.04 ± 1.25	39.12 ± 1.13	38.89 ± 1.41	1.10	0.334
Mode of delivery, [SVD *n* (%)]	59 (45.4%)	51 (39.2%)	61 (46.9%)	1.750	0.417
Age at enrollment (month)	22.75 ± 6.93	23.32 ± 7.43	22.39 ± 6.47	0.591	0.554
Feeding-history				0.598	0.741
Exclusively formula-fed from birth (*n*)	120	123	122		
Previously breastfed but fully weaned for at least 3 months before enrollment (*n*)	10	7	8		
Parental education level ^a^				2.560	0.862
Junior high school and below	44	42	45		
Senior high school/Technical secondary school	38	44	33		
Junior College/Vocational College	29	27	33		
Bachelor’s degree or above	19	18	21		
Household per capita income				16.915	0.076
< 2000RMB¥	5	6	1		
2001–3000RMB¥	26	29	16		
3,001–4000RMB¥	49	51	43		
4,001–5,000 RMB¥	24	20	40		
5,001–6,000 RMB¥	13	12	15		
> 6,001 RMB¥	11	10	13		
Registered residence [urban, *n* (%)]	21 (16.2%)	20 (15.4%)	21 (16.1%)	0.038	0.981
Number of household residents [*n* < 4, *n* (%)]	12 (9.2%)	12 (9.2%)	13 (10.0%)	0.060	0.970

^a^selecting the highest educated level of parents as the statistical data. SD, standard deviation; CG, the placebo control group; PG, the probiotic intervention group; HPG, the combination of 2′-fucosyllactose and probiotic intervention group.

### 3.2 Effects of IPs on the primary outcome

The incidence of URTIs over the 6-month study period differed significantly among the three groups [incidence in CG, PG, and HPG: 85.4% (111/130), 70.8% (92/130), and 55.4% (72/130), respectively; *χ^2^* = 28.15, *p* < 0.001] according to the ITT analysis. PP analyses yielded consistent results, with significant difference among the three groups [incidence in CG, PG, and HPG: 91.7% (111/121), 77.3% (92/119), and 59.0% (72/122), respectively; *χ^2^* = 35.80, *p* < 0.001]. These findings indicated that probiotics intervention resulted in statistically significant reduction in URTIs incidence, and the results were consistent across both ITT and PP analyses.

### 3.3 Effect of IPs on the episode days of common pediatric conditions

**Figure 2:**
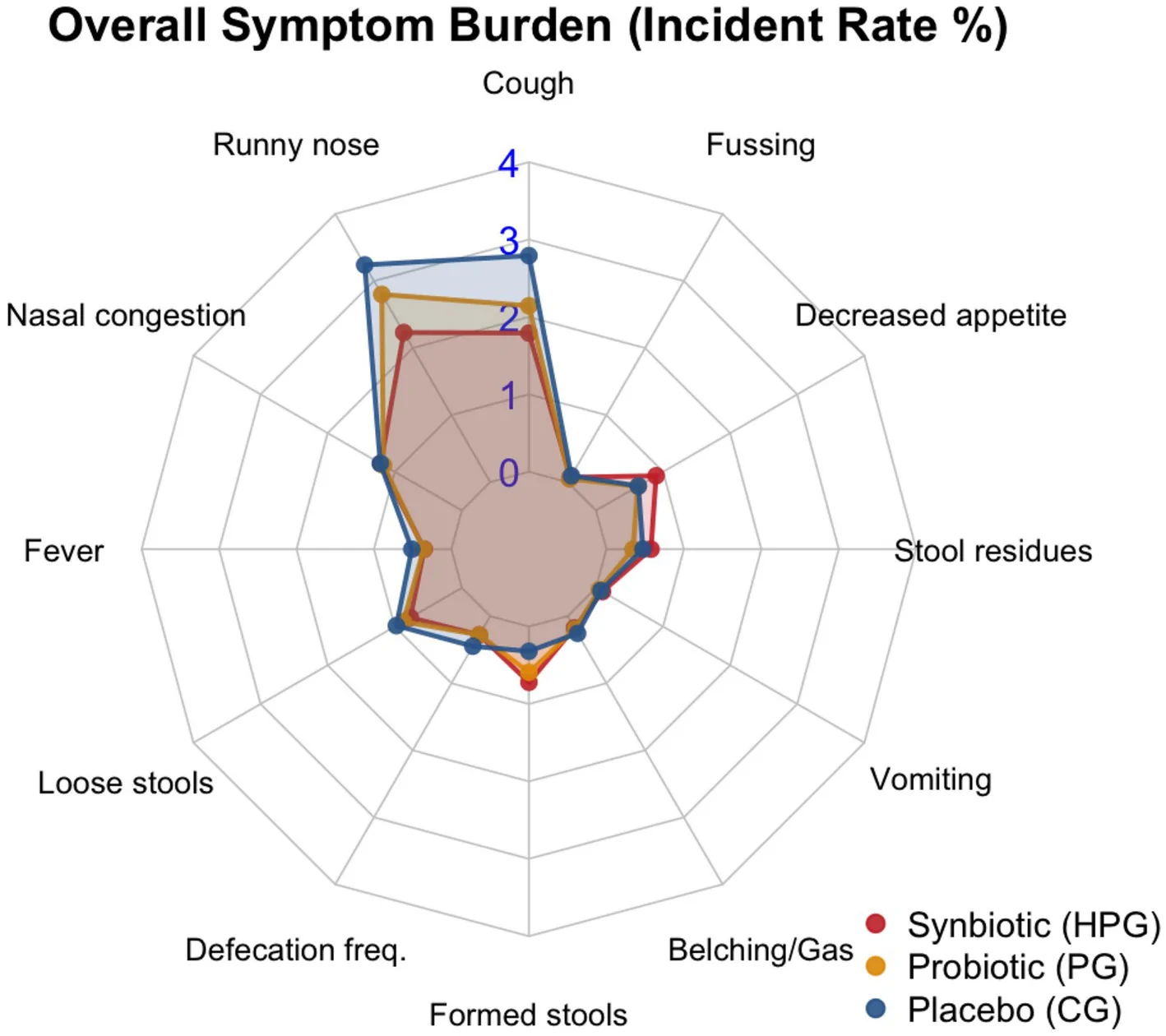
Radar chart of overall symptom burden among the three intervention groups. The overall symptom burden, as visualized in the radar chart, demonstrated a distinct reduction in incident rates among participants in the synbiotic (HPG) group compared to the CG group and PG group. CG, the placebo control group; PG, the probiotic intervention group; HPG, the combination of 2′-fucosyllactose and probiotic intervention group.

During the 6-month study period, total occurrence days of common respiratory, gastrointestinal, and skin symptoms, as well as incident rate per 100 intervention days and inter-group comparisons, are presented in Table 2 and Figure 2. Significant differences among the three groups were observed in total occurrence days of coughing, runny nose, fever, increased defecation frequency, formed stools, pneumonia, diarrhea and the days of antibiotic use (all *p* values < 0.05). *Kruskal-Wallis* nonparametric analysis indicated that, compared with children in the CG, children in the HPG experienced significantly fewer total occurrence days of coughing, runny nose, fever, increased defecation frequency, pneumonia, diarrhea and the days of antibiotic use (all *p* values < 0.05). Also, the occurrence of formed stools was significantly higher in the HPG than that of children in the CG (*p* < 0.05). However, PG group did not show any difference in the duration of common symptoms when compared with CG group.

**Table 2:** Comparison of the duration of common symptoms in children during the study period.

Common symptoms	HPG (*n* = 130)		PG (*n* = 130)		CG (*n* = 130)	
	Total occurrence days	Incident rate per 100 intervention days (%)^†^	Total occurrence days	Incident rate per 100 intervention days (%)^†^	Total occurrence days	Incident rate per 100 intervention days (%)^†^
Cough^*^	525	2.24a	626	2.68ab	816	3.49b
Runny nose^*^	654	2.79a	819	3.50ab	947	4.05b
Nasal congestion	353	1.51a	342	1.46a	355	1.52a
Fever^*^	104	0.44a	106	0.45ab	150	0.64b
Loose stools	225	0.96a	255	1.09a	286	1.22a
Increased defecation frequency ^*§^	82	0.35a	83	0.35ab	132	0.56b
Formed stools^*‡^	210	0.90a	173	0.74ab	94	0.4b
Increased belching/bloating/anal gas	51	0.22a	57	0.24a	76	0.32a
Retching/Vomiting	27	0.12a	14	0.06a	20	0.09a
Stool with milk flaps/food residues/sour odor	168	0.72a	100	0.43a	138	0.59a
Decreased appetite^&^	263	1.12a	183	0.78a	184	0.79a
Fussing	20	0.09a	13	0.06a	28	0.12a
URTIs	320	1.37a	417	1.78a	431	1.84a
Tracheitis/Bronchitis	166	0.71a	146	0.62a	202	0.86a
Pneumonia^*^	71	0.30a	130	0.56ab	230	0.98b
Diarrhea^*^	73	0.31a	82	0.35ab	124	0.53b
Daily crying episodes	179	0.76a	171	0.73aa	176	0.75a
Daily crying duration	1,341	5.73a	1,628	6.96a	1,394	5.96a
Average nighttime sleep duration	1,319	5.64a	1,329	5.68a	1,287	5.5a
Number of night awakenings	88	0.38a	109	0.47a	97	0.41a
Days of antibiotic use*	493	2.11a	610	2.61ab	817	3.49b

^†^A total of 23,400 study days (180 days × 130 people). Symptom incidence per 100 intervention days = duration of a symptom/total intervention days × 100%; ^§^Increased defecation frequency, daily frequency of defecation increased compared with that before the intervention as judged by caregivers/parents. ^*^Intergroup difference was statistically significant (*p* < 0.05 for all). ^&^Decreased appetite, daily food intake at least 50% lower than before the intervention as judged by the caregivers/parents. ^‡^Formed stool defined as Bristol Stool Form Scale types 3–5 stool Bristol score type. CG, the placebo control group; PG, the probiotic intervention group; HPG, the combination of 2′-fucosyllactose and probiotic intervention group. The symbols “a” and “b” indicate the results of the Kruskal–Wallis rank test for skewed data. Different letters represent statistically significant differences among groups, whereas identical letters indicate no statistically significant differences.

### 3.4 Effects of IPs on the morbidity of common pediatric conditions

**Figure 3:**
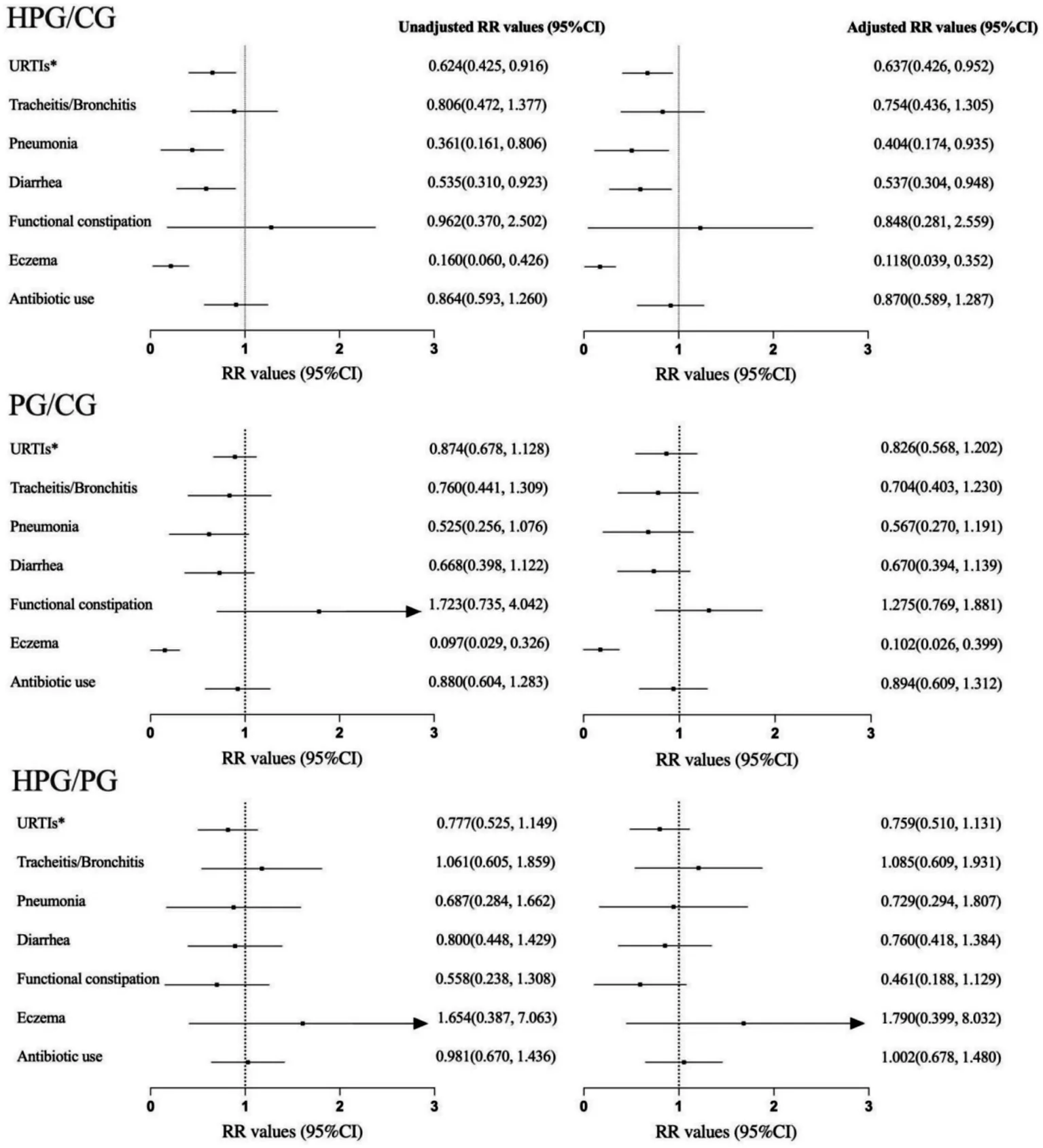
Effects of IPs on the risk ratios of common diseases and antibiotic use during the 6-month study period. URTIs: upper respiratory tract infection; RR, risk ratio; 95% CI, 95% confidence interval for the risk ratio; CG, the placebo control group; PG, the probiotic intervention group; HPG, the combination of 2′-fucosyllactose and probiotic intervention group. Adjusted RR, adjusting for the co-variate factors, including gender, household registration, ethnicity, mode of delivery, parents’ educational level, economic income, and number of family members.

Morbidity data were collected over a median duration of 180 days (interquartile range: 172–189 days) for three groups. The incidence of pneumonia in the CG, PG, and HPG was 17.7% (23/130), 9.2% (12/130), and 7.7% (10/130), respectively. The incidence of diarrhea was 17.7% (23/130), 13.1% (17/130), and 10.0% (13/130), respectively. The incidence of eczema was 13.1% (17/130), 2.3% (3/130), and 1.5% (2/130), respectively. Table 3 and Figure 3 summarized the incidences and the risk ratios (RR) and adjusted RR (aRR) for respiratory, gastrointestinal, and allergic diseases and antibiotic use during the study period.

**Table 3:** Adjusted relative risks (aRRs) of common childhood illnesses during the intervention period.

Outcome	HPG/CG		PG/CG		HPG/PG	
	aRR (95% CI)	*p*	aRR (95% CI)	*p*	aRR (95% CI)	*p*
URTIs^*^	0.637 (0.426, 0.952)	0.028	0.826 (0.568, 1.202)	0.318	0.759 (0.51, 1.131)	0.176
Tracheitis/Bronchitis	0.754 (0.436, 1.305)	0.313	0.70 4 (0.403, 1.23)	0.218	1.085 (0.609, 1.931)	0.782
Pneumonia	0.404 (0.174, 0.935)	0.034	0.567 (0.27, 1.191)	0.134	0.729 (0.294, 1.807)	0.495
Diarrhea	0.537 (0.304, 0.948)	0.032	0.67 (0.394, 1.139)	0.139	0.76 (0.418, 1.384)	0.37
Functional constipation	0.848 (0.281, 2.559)	0.769	1.275 (0.769, 1.881)	0.356	0.461 (0.188, 1.129)	0.09
Eczema	0.118 (0.039, 0.352)	< 0.001	0.102 (0.026, 0.399)	0.001	1.79 (0.399, 8.032)	0.447
Antibiotic use	0.87 (0.589, 1.287)	0.486	0.894 (0.609, 1.312)	0.566	1.002 (0.678, 1.48)	0.993

^*^URTIs: upper respiratory tract infection; RR, risk ratio; 95% CI, 95% confidence interval for the risk ratio; CG, the placebo control group; PG, the probiotic intervention group; HPG, the combination of 2′-fucosyllactose and probiotic intervention group. Adjusted RR, adjusting for the covariate factors, including gender, household registration, ethnicity, mode of delivery, parents’ educational level, economic income, and number of family members.

When compared with CG group, the RRs of the episodes of URTIs [RR and 95% confidence interval (95% CI): 0.624 (0.425, 0.916), *p* = 0.016], Pneumonia [RR and 95% CI: 0.361(0.161, 0.806), *p* = 0.013], Diarrhea [RR and 95% CI: 0.535(0.31, 0.923), *p* = 0.025], and Eczema [RR and 95% CI: 0.16(0.06, 0.426), *p* < 0.001] in HPG group were significantly lower; while only the RR of Eczema in PG group was significantly lower than that in CG group [RR (95% CI), 0.097(0.029, 0.326), *p* < 0.001]. However, there is no any difference between HPG group and PG group.

After adjusting for the bias factors of gender, household registration, ethnicity, mode of delivery, parents’ educational level, economic income, and number of family members, the results is consistent that, the aRRs of the episodes of URTIs [aRR and 95% CI: 0.637 (0.426, 0.952), *p* = 0.028], pneumonia [aRR (95% CI), 0.404 (0.174, 0.935), *p* = 0.034], diarrhea [aRR (95% CI), 0.537, (0.304, 0.948), *p* = 0.032], and eczema [aRR (95% CI), 0.118 (0.039, 0.352), *p* < 0.001] in HPG were significantly lower than those in CG group; while only the aRR of eczema [aRR (95% CI), 0.102 (0.026, 0.399), *p* = 0.001] in PG group was significantly lower than CG group. Notably, the aRR of functional constipation has a declining trend in HPG group than that in PG group, but with no statistical significance (*p* = 0.09).

All these results indicated that probiotics is protective factor that can decrease the morbidity of eczema. Besides, probiotics combined with HMO has additional benefit on preventing URTIs, Pneumonia and Diarrhea in healthy young children.

### 3.5 Effect of IPs on gut microbiota

#### 3.5.1 Effect of IPs on gut microbiota diversity

Alpha diversity was utilized to assess microbial community diversity within samples. QIIME software was employed to calculate the alpha diversity indices: observed species as the richness index, Shannon as the diversity index, Pielou_J as the evenness index and Pd_faith as the evolutionary diversity index. In the present data, there were no significant differences in any of the four alpha diversity indices among the three groups at baseline, 3-month intervention or 6-month follow-up (all *p* values > 0.05).

**Figure 4:**
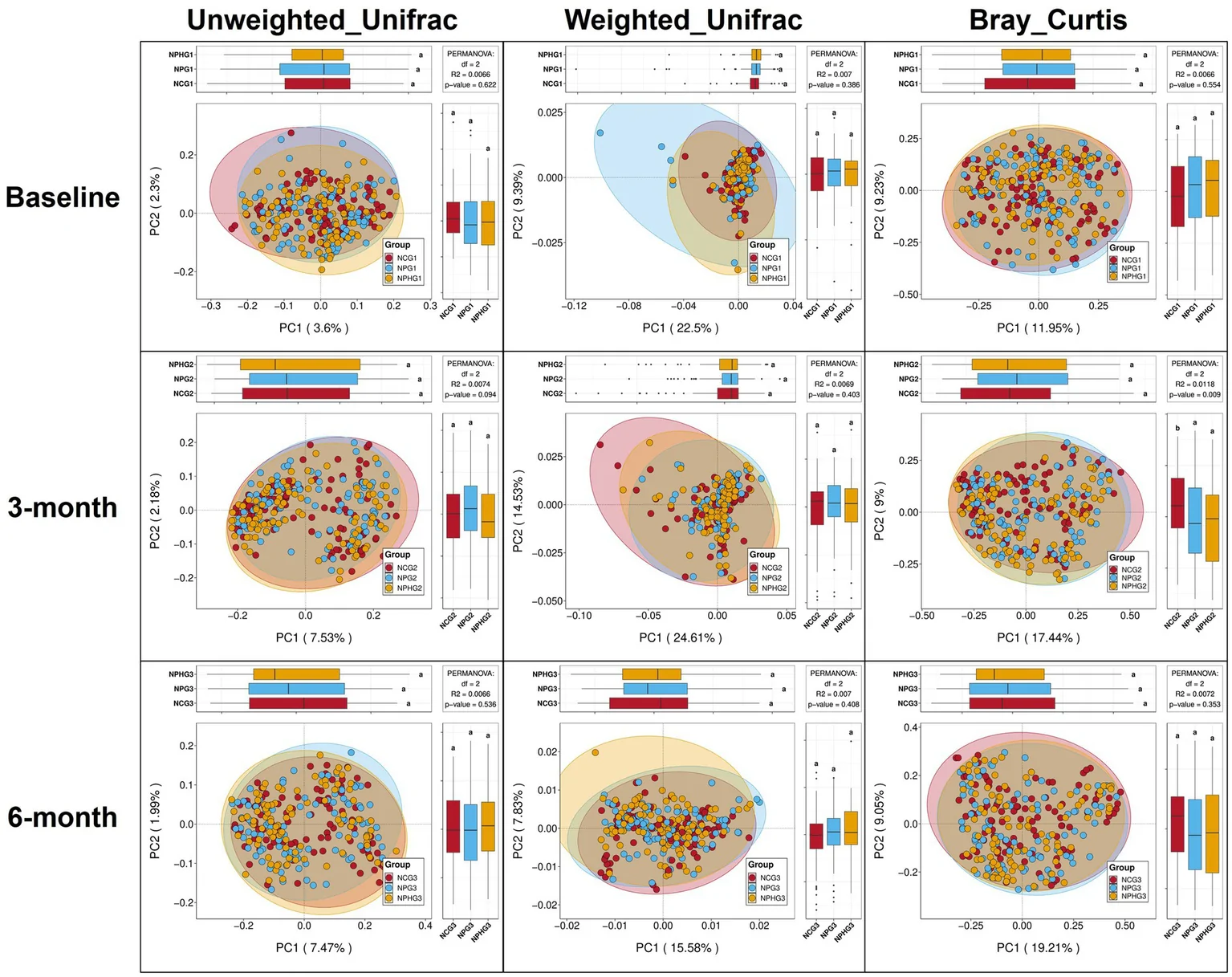
PCoA analysis of beta diversity among three groups before and after intervention calculated by three different distance metrics including unweighted_unifrac, weighted_unifrac, and bray_curtis. NCG, placebo control group (CG); NPG, probiotics group (PG); NPHG, 2’-FL and probiotic intervention group (HPG). Bars sharing the same letters indicate no significant difference between groups (*p* > 0.05); ars with different letters indicate statistically significant differences (*p* < 0.05).

PCoA plots based on three different distance metrics was presented in Figure 4. At baseline, there is no significant differences in PC1 and PC2 components among the three groups (*p* > 0.05). After 3 months intervention, HPG and PG group were significantly separated from CG group along the PC2 coordinate on Bray-Curtis metrics (*p* < 0.05), indicated there was trend of separation with HMO probiotics or probiotics intervention by gut microbiota, while no statistical difference between two interventional groups (*p* > 0.05). However, at 6 month follow-up time point, there is no significant difference in beta-diversity among the three groups (*p* > 0.05).

#### 3.5.2 Comparison of microbiome composition among groups

**Figure 5:**
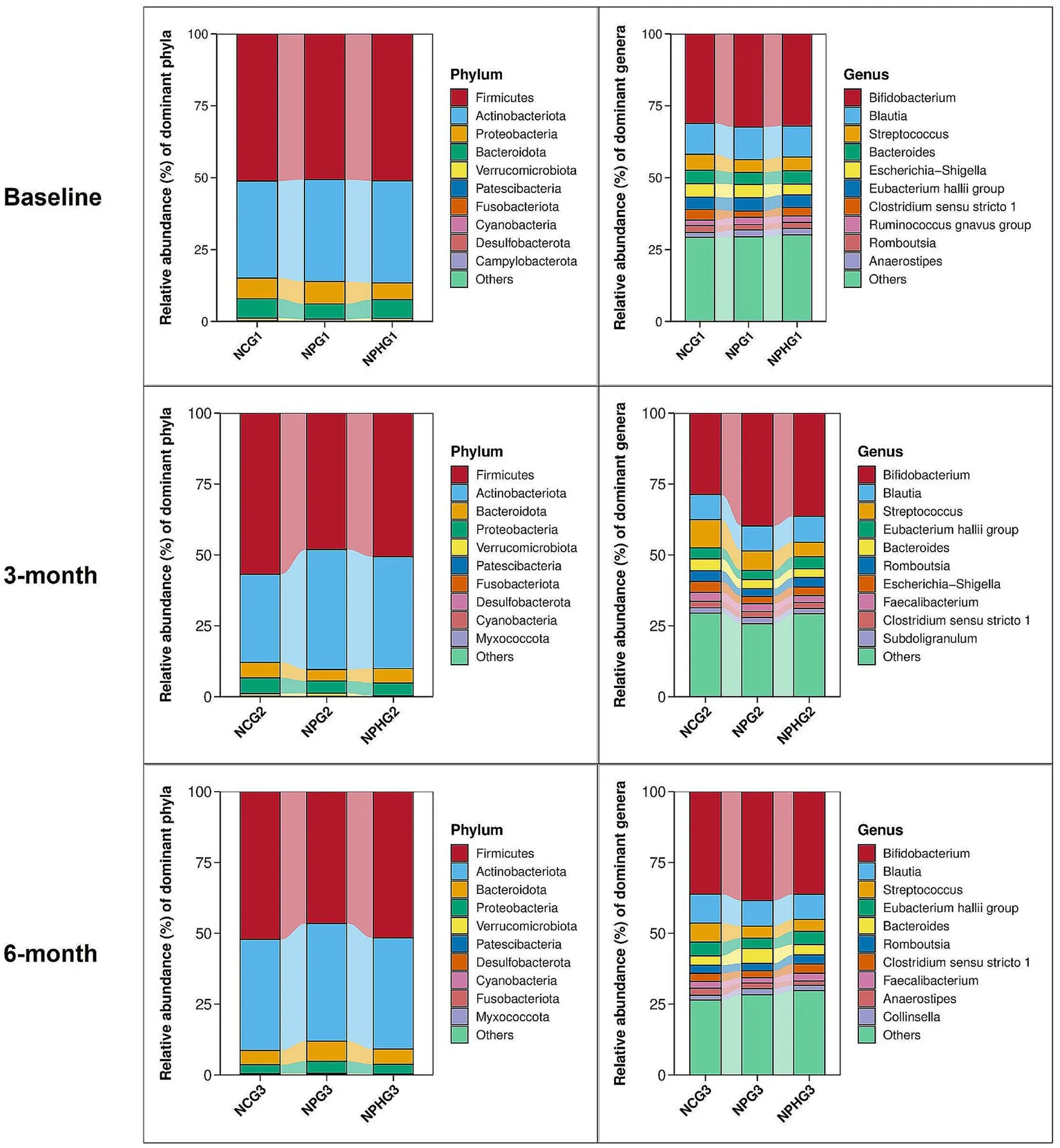
Comparison of the top 10 genus-level relative abundance differences among groups. NCG, placebo control group (CG); NPG, probiotics group (PG); NPHG, 2’-FL and probiotic intervention group (HPG). Bars sharing the same letters indicate no significant difference between groups (*p* > 0.05); Bars with different letters indicate statistically significant differences (*p* < 0.05).

The relative abundances of the top 10 genera and species across the three groups are shown in Figure 5. Throughout the clinical trial, at the phylum level, the fecal microbiota of the participants was predominantly composed of Firmicutes, Actinobacteriota, Bacteroidota, and Proteobacteria. At the genus level, the top 10 most abundant genera were *Bifidobacterium*, *Blautia*, *Streptococcus*, *Eubacterium hallii* group, *Bacteroides*, *Romboutsia*, *Clostridium sensu stricto* 1, *Faecalibacterium, Anaerostipes*, and *Collinsella*.

**Figure 6:**
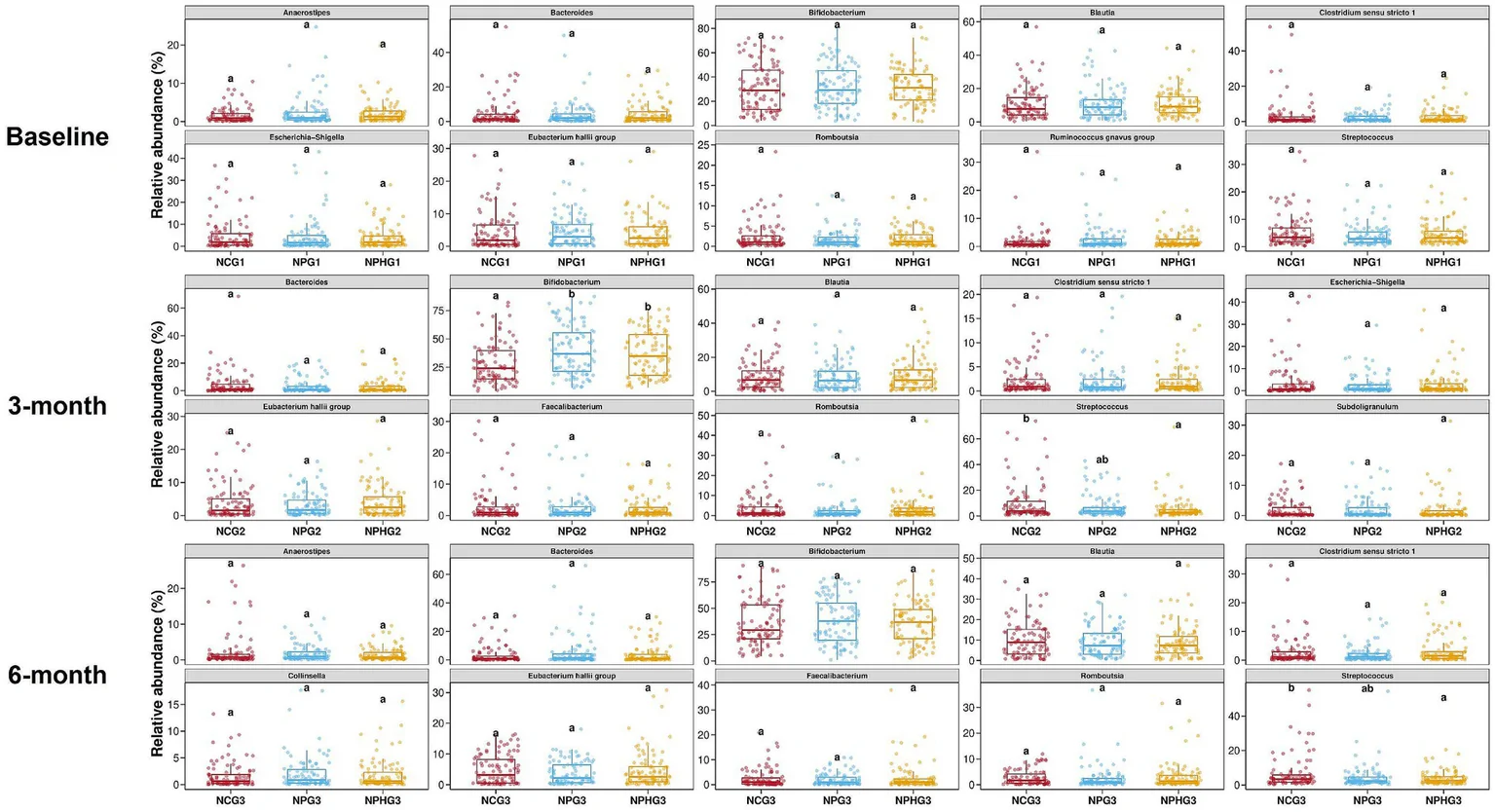
Comparison of the top 10 genus-level relative abundance differences among groups. NCG, placebo control group (CG); NPG, probiotics group (PG); NPHG, 2’-FL and probiotic intervention group (HPG). Bars sharing the same letters indicate no significant difference between groups (p > 0.05); bars with different letters indicate statistically significant differences (*p* < 0.05).

**Figure 7:**
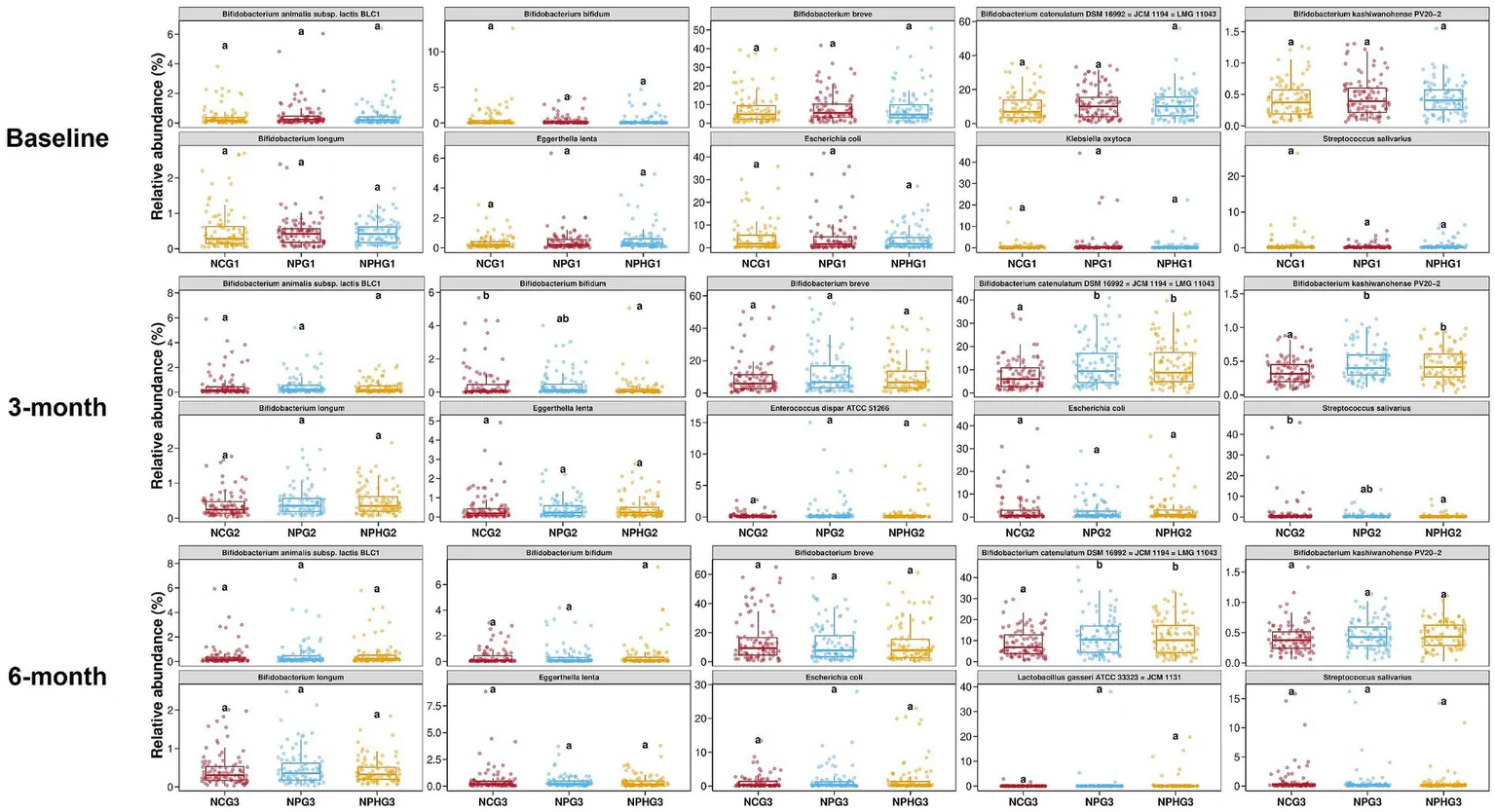
Comparison of the top 10 species-level relative abundance differences among groups. NCG, placebo control group (CG); NPG, probiotics group (PG); NPHG, 2’-FL and probiotic intervention group (HPG). Bars sharing the same letters indicate no significant difference between groups (*p* > 0.05); bars with different letters indicate statistically significant differences (*p* < 0.05).

Differential abundance analysis of the top 10 genera and species across the three groups at different time points are shown in Figures 6, 7. At baseline, no significant differences in genera or species were observed among the three groups. However, compared to the placebo group, the relative abundance of the *Bifidobacterium* genus was significantly increased after 3 months of intervention, but returned to baseline at 6-month follow-up. Meanwhile, the HPG group showed a significant reduction in the *Streptococcus* genus both after the 3-month intervention and 6-month follow-up, whereas PG showed a decreasing trend without statistical significance.

Further species-level analysis revealed that 3 months of intervention significantly enriched *B. catenulatum* DSM 16992 and *B. kashiwanohense* PV20-2 (Figure 7). Specifically, only the significantly increase in *B. catenulatum* DSM 16992 was remained at 6-month follow-up. Although a significant decrease in *B. bifidum* and *S. salivarius* was observed in the HPG group after the intervention, these differences disappeared across all three groups by the end of the follow-up period.

#### 3.5.3 Species identification of abundance differences between two pairwise groups

**Figure 8:**
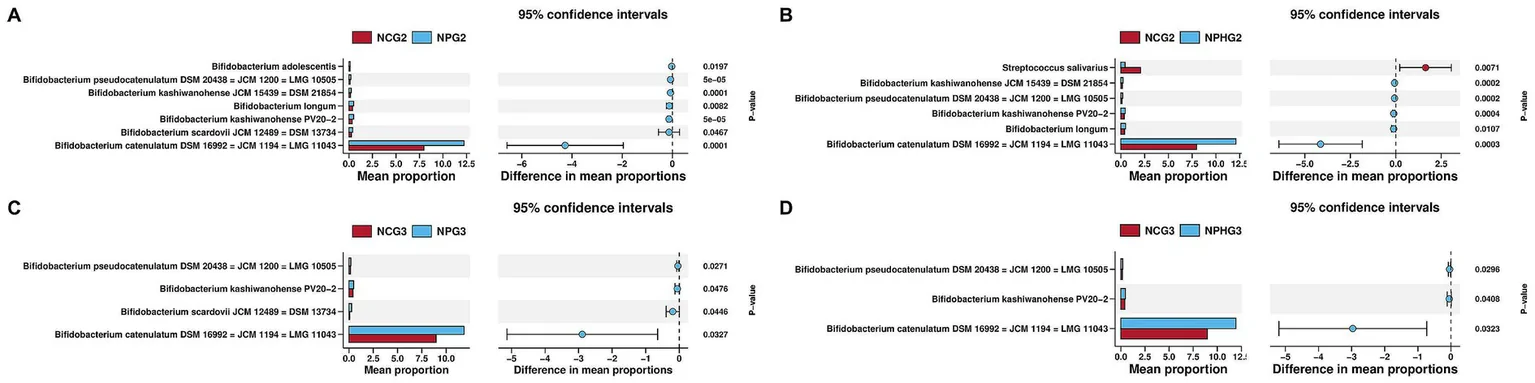
Species levels identification of abundance differences between two pairwise groups after 3-month intervention **(A,B)** and 6-month follow-up **(C,D)**. NCG, placebo control group (CG); NPG, probiotics group (PG); NPHG, 2’-FL and probiotic intervention group (HPG). After the intervention, no statistically significant differences in species levels were detected between the PG and the HPG, as well as among the three groups (CG, PG, and HPG) before the intervention, that met the preset screening criteria (i.e., detection rate ≥ 60% and average relative abundance of the entire sample ≥ 0.5%) and were corrected by the *Wilcoxon* rank sum test with *p* value < 0.05.

The *Wilcoxon* rank-sum test was employed to assess inter-group differences in species relative abundance between the two pair each group (Figure 8). Prior to statistical testing, a species filtering step was applied: only species detected in ≥60% of samples within at least one group and exhibiting a mean relative abundance ≥0.5% across all samples were retained for analysis. The present data showed that 3-month intervention with HPG significantly increased the relative abundance of *B. pseudocatenulatum* DSM 20438, *B. longum, B. kashiwanohense* JCM 15439, *B. kashiwanohense* PV20-2, and *B. catenulatum* DSM 16992 (Figures 8A,B) when compared to the CG (*p* values < 0.05). Besides, PG significantly enriched *B. adolescentis* and *B. scardovii* JCM 12489 = DSM 13734, and HPG could significantly decrease *S. salivarius* (*p* values < 0.05).

At 6-month follow-up, HPG and PG still significantly enriched *B. pseudocatenulatum* DSM 20438, *B. kashiwanohense* PV20-2, and *B. catenulatum* DSM 16992, which means all the *Bifidobacterium* species enriched by HPG and PG could colonize and survive until 3 month after intervention.

### 3.6 Effect of IPs on fecal biochemical index

**Figure 9:**
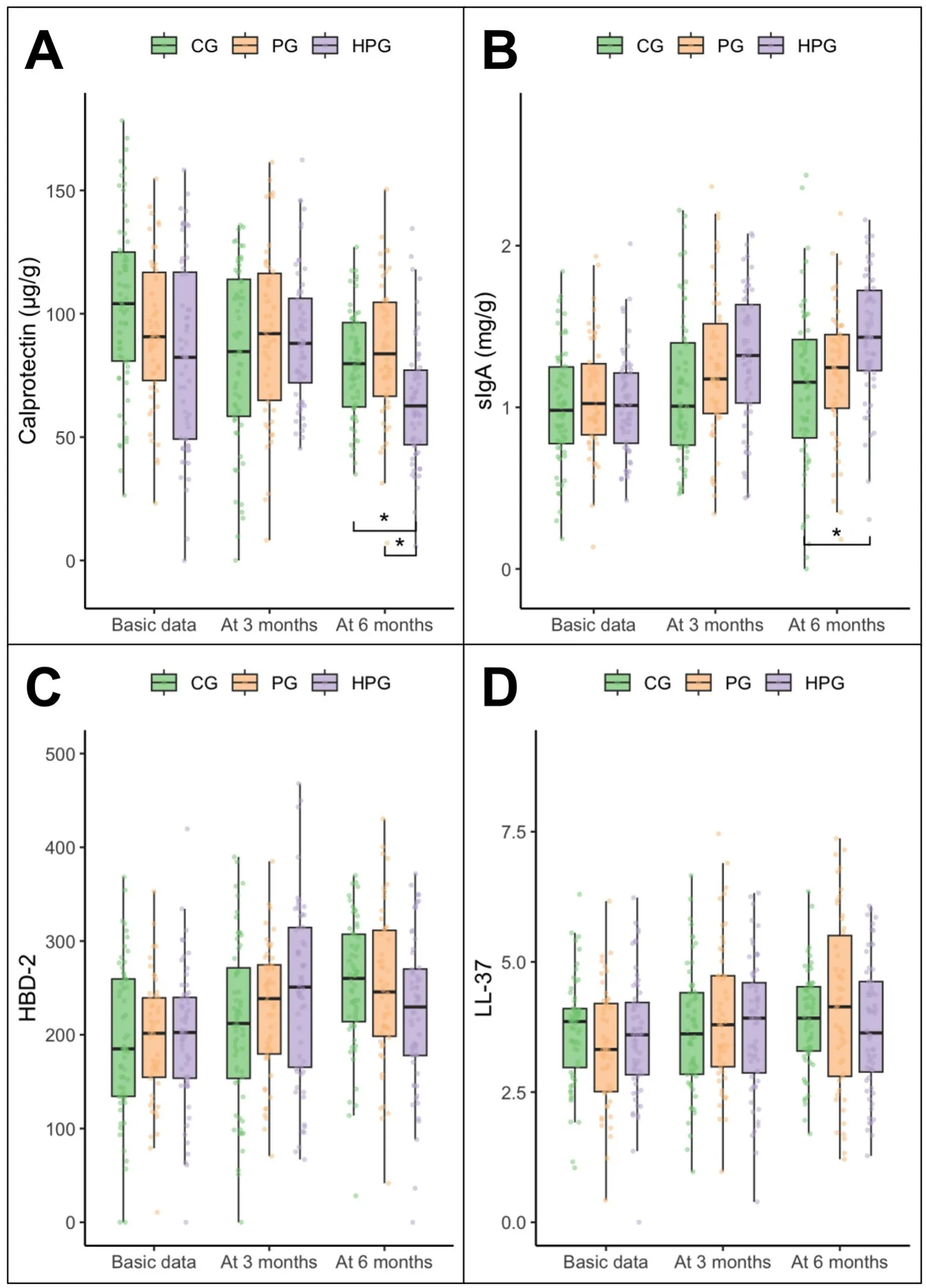
Effect of intervention products on fecal biochemical index. **(A)** calprotectin; **(B)** ‌secretory immunoglobulin A (sIgA); **(C)** ‌human *β*-defensin 2 (HBD-2); **(D)** ‌human cathelicidin LL-37 (LL-37). CG, placebo control group; PG, probiotics intervention group; HPG, 2’-FL and probiotic intervention group; *, difference with statistical significance (*p* < 0.0167).

At baseline and 3 months after intervention, there were no significant difference among the three groups in fecal levels of calprotectin, sIgA, HBD-2 and LL-37 (all *p values* > 0.05, Table 4, Figure 9). However, at the 6 months follow-up, HPG intervention significantly reduced the calprotection level [62.65 ± 23.94 vs. 74.70 ± 30.20 vs. 77.94 ± 24.59 μg/g for HPG, PG and CG, respectively, *p* = 0.004], and elevated the sIgA level [1.37 ± 0.38 vs. 1.24 ± 0.38 vs. 1.15 ± 0.49 μg/g for HPG, PG and CG, respectively, *p* = 0.014] when compared with CG and PG group. However, no significant difference was found for HBD-2 or LL-37 levels among the groups at 6 month follow-up (all *p* values > 0.05).

**Table 4:** Effects of IPs on fecal biochemical indexes in children during the intervention period (mean ± standard deviation).

Fecal biochemical indicators		CG (n = 60)	PG (*n* = 50)	HPG (*n* = 60)	*F* values	*p* values
Calprotectin (μg/g)	Basic data^*^	97.37 ± 35.5a	92.58 ± 30.8a	89.36 ± 38.9a	0.785	0.458
	At 3 months^*^	79.58 ± 40.3a	90.37 ± 34.5a	84.08 ± 29.0a	1.447	0.238
	At 6 months^*^	77.94 ± 24.6a	74.70 ± 30.2a	62.65 ± 23.9b	5.592	0.004
sIgA (mg/g)	Basic data^*^	0.973 ± 0.4a	1.06 ± 0.4a	1.04 ± 0.3a	1.108	0.332
	At 3 months^*^	1.12 ± 0.5a	1.22 ± 0.5a	1.26 ± 0.4a	1.351	0.262
	At 6 months^*^	1.15 ± 0.5a	1.24 ± 0.4ab	1.37 ± 0.4b	4.397	0.014
HBD-2 (pg/g)	Basic data^*^	201 0.19 ± 95.0a	191.09 ± 68.9a	202.64 ± 71.1a	0.378	0.686
	At 3 months^*^	226.79 ± 79.6a	234.1 7 ± 75.8a	240.94 ± 90.8a	0.443	0.643
	At 6 months^*^	239.38 ± 70.5a	233.09 ± 86.2a	232.42 ± 87.9a	0.132	0.876
LL-37 (ng/g)	Basic data^*^	3.61 ± 1.1a	3.42 ± 1.2a	3.40 ± 1.3a	0.576	0.563
	At 3 months^*^	3.66 ± 1.2a	3.80 ± 1.3a	3.75 ± 1.2a	0.196	0.822
	At 6 months^*^	3.73 ± 0.9a	3.86 ± 1.7a	3.56 ± 1.4a	0.721	0.488

^*^Student–Newman–Keuls test among the three groups: identical letters indicate no statistically significant differences among groups (*p* > 0.0167), while different letters indicate a statistically significant difference among groups (*p* < 0.0167); IPs, intervention productions; CG, placebo control group; PG, probiotics intervention group; HPG, 2’-FL and probiotic intervention group; sIgA‌, ‌secretory immunoglobulin A; HBD-2‌, ‌human β-defensin 2; LL-37, ‌human cathelicidin LL-37‌. The symbols “a” and “b” indicate the results of the Student–Newman–Keuls test among the three groups. Identical letters indicate no statistically significant differences among groups (*p* > 0.0167), whereas different letters indicate statistically significant differences among groups (*p* < 0.0167).

## 4 Discussion

To our knowledge, there is a scarcity of randomized controlled trials (RCTs) that directly compare the efficacy of a probiotic-only intervention against a targeted synbiotic intervention comprising the same probiotic strains and a structurally defined HMO, like 2’-FL, in a toddler population. Most researches only focused on special probiotics alone, or prebiotics alone (often galacto-oligosaccharides [GOS] and fructo-oligosaccharides [FOS]), or synbiotic combinations that the prebiotic component is not a specific HMOs. The present study is therefore addressing a critical question that whether an addition of a specific, bioactive HMO can enhance the protective effects of well-characterized probiotic strains.

In this multi-center RCT involving children aged 1–3 years, 3-month supplementation with a dual-strain probiotic (*B. infantis* R0033 and *B. bifidum* R0071), with or without 2’-FL, significantly reduced the burden of common pediatric conditions throughout the 6-month observation period. Consistent with previous studies, these specific strains classified as infant-type human-residential Bifidobacteria (HRB) had been reported to confer clinical benefits in infants, including promoting intestinal maturation, enhancing mucosal immunity, and preventing common pediatric infections (27, 28). Besides, recent pediatric reviews have highlighted that *Bifidobacterium*-dominant probiotics are associated with reduced RR of atopic dermatitis and selected infections when administered in adequate doses (29, 30). Taken together, these findings support the biological plausibility of the protective effects observed in our study.

Compared with the placebo, the HMO probiotics group had significantly lower adjusted RRs of URTIs, pneumonia, diarrhea, and eczema, as well as fewer total symptom-days for several respiratory and gastrointestinal manifestations and fewer antibiotic-use days. Notably, the incidence of URTIs was lowest in the HPG (55.4%), compared with 70.8% in the PG and 85.4% in placebo, corresponding to a 36.3% lower risk relative to placebo. Although differences between HPG and PG did not reach statistical significance in all adjusted head-to-head comparisons, the HPG showed a consistent pattern of greater effect magnitude across most outcomes. Although direct comparisons between HPG and PG did not reach statistical significance, HPG consistently showed numerically lower disease risks and symptom burden across several outcomes. These findings may suggest a potential contribution of 2’-FL; however, the present study was not specifically powered to detect differences between the two active intervention groups, and therefore this interpretation should be considered exploratory. Extensive clinical evidence has demonstrated that the addition of HMOs, such as 2’-FL and lacto-N-neotetraose (LNnT), to infant formula can effectively modulate the gut microbiota, shifting its composition closer to that of breast-fed infants, contributing to a reduced risk of respiratory tract infections later in early childhood (31–33). These modifications are primarily characterized by a significant increase in the relative abundance of HMO-utilizing bifidobacteria (e.g., *B. infantis*, *B. breve*) and a parallel reduction in potential pathogens like *Escherichia* and *Klebsiella* (31, 34). Beyond gut microbiota regulation, 2’-FL could be served as soluble decoy receptors that inhibit the adhesion of pathogens to the intestinal epithelium, including *C. jejuni*, enteropathogenic *E. coli*, *Salmonella enterica*, rotavirus, norovirus, and respiratory syncytial virus (35). Clinically, the broader disease reduction spectrum observed in the HPG supports the role for intestinal homeostasis in multiple mechanisms, including respiratory defense via the gut–lung axis, improved intestinal barrier function and pathogen resistance, and immune tolerance pathways relevant to eczema risk. Collectively, these findings support a synergistic interaction between HMOs and bifidobacterial probiotics, consistent with the concept of rationally designed synbiotic interventions.

In this clinical study, microbiota analyses indicated that probiotic-containing interventions increased the relative abundance of *Bifidobacterium* genus compared with placebo, especially *B. catenulatum*, which are most commonly present in adults (36). Previous whole-genome analyses have shown that *B. catenulatum* possesses the genetic capacity to metabolize 2’-FL (37); thus, the 2’-FL-enriched HPG directly provides an essential carbon source for its growth. In contrast, the enrichment observed in the PG may be attributed to microbial cross-feeding. For instance, extracellular hydrolases from other species, such as *B. bifidum*, can liberate terminal sugar moieties from mucin, thereby providing supplementary carbon sources for *B. catenulatum* (38). Despite a previous study linking 3-month-old colonization of *B. catenulatum* to higher eczema risk, the clinical significance of this species in older children is not yet fully elucidated (39). Given that *B. catenulatum* has been shown in animal models to facilitate *F. prausnitzii* proliferation and SCFA production through cross-feeding, exerting synergistic anti-inflammatory effects (40). Therefore, the HPG and PG groups may enrich *B. catenulatum*, which in turn helps maintain gut microbial homeostasis and immune balance via cross-feeding mechanisms.

Fecal calprotectin is widely recognized as a sensitive, noninvasive biomarker of intestinal inflammation, reflecting neutrophil migration into the gut lumen and mucosal immune activation (41, 42). Although all groups demonstrated a decline in calprotectin over time, likely reflecting physiological maturation, the reduction was significantly greater in HPG at 6 months. This finding suggests that HMO probiotics supplementation can accelerate the normalization of intestinal inflammatory status in early childhood. Recent pediatric trials have reported the reductions in fecal calprotectin following synbiotic administration in children with functional gastrointestinal disorders or mild inflammatory conditions (43). Moreover, studies examining formulas supplemented with 2’-FL have demonstrated lower systemic and intestinal inflammatory markers, approximating profiles observed in breastfed infants (19). The enhanced anti-inflammatory effect observed in HPG compared with PG likely reflects this substrate-driven amplification of probiotic function, supporting the concept of true synbiotic synergy rather than additive effects alone.

Secretory IgA is a cornerstone of intestinal adaptive immunity, playing a critical role in immune exclusion, pathogen neutralization, and maintenance of microbial homeostasis. In the present study, fecal sIgA concentration was the highest in HPG at 6 months, significantly exceeding that in CG and numerically surpassing PG. This finding indicates that HMO probiotic intervention effectively promotes mucosal immune maturation. Recent clinical studies suggest that HMOs can modulate dendritic cell function, enhance regulatory T-cell responses, and stimulate IgA-secreting plasma cell differentiation (15, 44). When combined with probiotics, HMOs may potentiate probiotic-driven immune signaling by increasing bacterial abundance and metabolic outputs, thereby amplifying downstream IgA responses. Higher mucosal sIgA levels are associated with improved resistance to respiratory and gastrointestinal infections and better maintenance of intestinal barrier integrity (45–47). The simultaneous reduction in fecal calprotectin and increase in sIgA should not be interpreted as opposing immune responses. Rather, these biomarkers reflect distinct aspects of mucosal immunity. Calprotectin primarily indicates inflammatory activity and neutrophil recruitment, whereas sIgA reflects adaptive mucosal immune competence and immune exclusion. Therefore, the observed pattern is consistent with improved intestinal immune homeostasis, characterized by reduced inflammatory burden together with enhanced barrier-associated immune protection.

Early childhood represents a critical window for immune programming. Persistent low-grade intestinal inflammation has been associated with increased susceptibility to infections and allergic diseases later in life. Our findings suggest that synbiotic supplementation with 2’-FL and dual Bifidobacterium strains may facilitate immune maturation and promote a more balanced intestinal immune environment. Compared with probiotics alone, the addition of 2’-FL yielded superior outcomes in both inflammatory and adaptive immune markers. These results provide clinical evidence reinforcing the concept that combining HMOs with specific probiotic strains may more closely approximate the immunological benefits of breastfeeding.

Although caregivers were instructed to administer the intervention products and antibiotics at least 3 h apart, antibiotic exposure during the study may have reduced probiotic survival and transient gut colonization. Because antibiotic treatment was permitted for ethical and clinical reasons and represented part of routine pediatric care, participants receiving antibiotics were retained in the intention-to-treat analyses. As antibiotic exposure was balanced among the randomized groups, its influence is unlikely to have introduced systematic bias; however, it may have attenuated the magnitude of microbiota-related intervention effects.

Exploratory sex-stratified analyses did not reveal any apparent sex-related differences in the clinical, microbiological, or immune outcomes; however, the study was not specifically powered to detect sex-treatment interactions.

## 5 Study limitations

Several limitations about the present study should be considered. Firstly, the study population was drawn from defined community settings within a single regional context. Environmental exposures, dietary patterns, and baseline microbiota profiles may differ across populations, which may affect external generalizability. In addition, although all enrolled children were either exclusively formula-fed or had been fully weaned for at least 3 months before enrollment, detailed information regarding the duration of previous breastfeeding exposure was not systematically collected. Since breastfeeding may exert long-term effects on immune maturation and gut microbiota development, residual confounding associated with early feeding history cannot be completely excluded. Future studies should collect detailed breastfeeding data and perform stratified analyses according to feeding history. Secondly, the intervention period was three months with three additional months of follow-up. While sufficient to detect differences in common illness incidence, longer-term studies are needed to determine persistence of microbiota changes and durability of clinical protection. Thirdly, although disease diagnoses followed clinical guidelines and were recorded in standardized CRF, some symptom measures relied on caregiver reporting. Reporting variability cannot be fully excluded in community-based pediatric trials. Fourthly, as outlined in the initial study design, the sample size was calculated based on the relatively high clinical incidence of URTIs. Consequently, the statistical power may have been insufficient to detect significant effects for diseases with lower incidence rates or for certain fecal 16sRNA indicators. Lastly, administering a single dose of 1.5 billion CFU/day each of *B. infantis* R0033 and *B. bifidum* R0071 limited our ability to investigate the optimal dose–response relationship of these two strains in preventing respiratory, gastrointestinal, and allergic diseases.

## 6 Conclusion

In children aged 1–3 years old, supplementation with a dual-strain *Bifidobacterium* probiotic was associated with reduced disease burden, particularly for eczema. The combination of probiotics with 2′-fucosyllactose produced broader and stronger protective associations, including significant reductions in URTIs, pneumonia, diarrhea, and eczema, together with fewer symptom-days and reduced antibiotic use. These findings are consistent with current pediatric microbiome and nutrition research indicating that microbiota-directed interventions can contribute to common diseases risk reduction in early childhood. Probiotics alone were associated with beneficial clinical effects, while the combination of probiotics and 2’-FL showed a consistent trend toward broader protective effects. However, larger studies are needed to determine whether the addition of 2’-FL confers benefits beyond those achieved with probiotics alone. Synbiotic strategies incorporating HMOs may therefore represent a promising preventive approach for common childhood illnesses and warrant further long-term pediatric investigation.

## Data Availability

Raw 16SrRNA gene sequences for all fecal samples used in this study have been deposited in the National Center for Biotechnology Information BioProject database with the BioProject ID PRJNA1366446 (https://www.ncbi.nlm.nih.gov/bioproject/PRJNA1366446).

## Footnotes

1
https://www.chictr.org.cn/showproj.html?proj=235745
