# Correction: Precision synbiotic intervention with 2'-fucosyllactose and infant-derived *Bifidobacterium* modulates gut-immune axis and reduces disease risk in toddlers: a randomized controlled trial

**DOI:** 10.3389/fnut.2026.1957323

**Published:** 2026-09-16

**Authors:** Ke Chen, Xi Zhang, Shanshan Jin, Jiayi Zhong, Haixia Chen, Genquan Yue, Yonghui Cheng, Rufeng Peng, Ziji Fang, Feitong Liu, Zerong Lu, Lingling Zhao, Ruibiao Hu, Xiaohan Tao, Qilin He, Changqi Liu

**Affiliations:** 1 Department of Clinical Nutrition, Chengdu Women's & Children's Central Hospital, School of Medicine, University of Electronic Science and Technology of China, Chengdu, China; 2 Baoxing Center for Disease Control and Prevention, Ya'an, China; 3 Longmen Town Central Health Center, Ya'an, China; 4 Baoxing Maternal and Child Health Care Hospital, Ya'an, China; 5 School of Medicine, University of Electronic Science and Technology of China, Chengdu, China; 6 H&H Group, China Research and Innovation Center, Guangzhou, China; 7 School of Food Science and Engineering, South China University of Technology, Guangzhou, China; 8 Graduate School of International Business, the Catholic University of Korea, Bucheon, Republic of Korea; 9 School of Exercise and Nutritional Sciences, San Diego State University, San Diego, CA, United States

**Keywords:** 2′-fucosyllactose, *Bifidobacterium*, gut microbiota, mucosal immunity, synbiotics

A correction has been made to the **Abstract:** “**Methods:** In this multicenter, double-blind, placebo-controlled, three-arm trial, 390 healthy toddlers were randomized (1:1:1) to receive (1) synbiotic (2′-FL 1.0 g/day + *B. infantis* R0033 1.5 × 10^9^ CFU + *B. bifidum* R0071 1.5 × 10^9^ CFU);”

A correction has been made to the **Section 2**, *Section 2.4*, paragraph one:

“All eligible children were randomly assigned to the HPG, IG or CG. The children in HPG received a daily supplement of 1.5 billion CFU each of *B. infantis* R0033 and *B. bifidum* R0071 and 1.0 g of 2′-FL (Production No: 87-BCP-20027).”

A correction has been made to the **Section 5**, paragraph one:

“Lastly, administering a single dose of 1.5 billion CFU/day each of *B. infantis* R0033 and *B. bifidum* R0071 limited our ability to investigate the optimal dose–response relationship of these two strains in preventing respiratory, gastrointestinal, and allergic diseases.”

The original version of this article has been updated.

